# A lnc6100-Mediated ceRNA Network Regulates Dopamine-Dependent Food Wanting in Honeybees

**DOI:** 10.3390/biom16101452

**Published:** 2026-10-05

**Authors:** Zun Wu, Ying Zhu, Hangyu Zhang, Rong Li, Chuanbao Jiang, Lingling He, Xueling Xu, Songkun Su

**Affiliations:** 1 College of Bee Science, Fujian Agriculture and Forestry University, Fuzhou 350002, China; 2 College of Animal Science, Fujian Agriculture and Forestry University, Fuzhou 350002, China

**Keywords:** *Apis mellifera*, food wanting, lncRNA, miR-375-3p, dopamine

## Abstract

The honeybee, *Apis mellifera*, is a highly social and ecologically important pollinator whose foraging activity is essential for colony nutrition and survival. Food wanting is a fundamental motivational state that drives food-seeking and foraging behaviors, yet the molecular mechanisms underlying dopamine-dependent food motivation remain poorly understood. Here, we integrated transcriptomic profiling of honeybee brains (*n* = 120) with bioinformatic prediction to identify three candidate lncRNAs highly associated with miR-375-3p, a microRNA previously implicated in the regulation of food wanting through the dopamine synthesis pathway. Functional screening using three complementary behavioral assays then identified lnc6100 as a key regulator of food wanting. Mechanistically, dual-luciferase reporter assays confirmed that lnc6100 directly interacts with miR-375-3p. Knockdown of lnc6100 increased miR-375-3p expression, reduced *Ddc* expression, and decreased brain dopamine levels, indicating that lnc6100 promotes dopamine synthesis through the miR-375-3p/*Ddc* pathway. Furthermore, inhibition of miR-375-3p rescued the impaired food wanting, restored *Ddc* expression, and increased dopamine levels in lnc6100-knockdown honeybees. Together, these findings support an lnc6100–miR-375-3p–*Ddc* regulatory axis through which noncoding RNA interactions regulate dopamine synthesis and food wanting in honeybees. Our study reveals the molecular mechanism by which the upstream noncoding RNA that regulates dopamine-dependent food wanting, providing new insights into lncRNA-based strategies to improve honeybee colony health.

## 1. Introduction

Honeybees are eusocial insects of considerable ecological and economic importance worldwide, playing indispensable roles in maintaining ecosystem stability and providing pollination services for agricultural crops [1]. Foraging by worker bees constitutes the primary means of resource acquisition for the colony, and its efficiency directly determines food supply, colony growth, reproductive success, and environmental adaptability [2]. Food wanting, defined as the internal motivational state that drives animals to actively seek and obtain food, represents a fundamental motivational component of foraging behavior [3]. In recent years, multiple environmental stressors, including pesticide exposure, pathogen infection, nutritional imbalance, and habitat degradation, have been shown to impair honeybee foraging activity and are considered important contributors to colony decline [4,5]. Therefore, elucidating the molecular mechanisms underlying the regulation of food wanting in honeybees will not only advance our understanding of the neurobiological basis of behavioral plasticity in honeybee foraging, but also provide new insights into the molecular mechanisms by which environmental stressors compromise colony health, thereby facilitating the development of potential intervention strategies.

Dopamine (DA) is an evolutionarily conserved biogenic amine neurotransmitter that plays central roles in regulating a wide range of complex behaviors, including motivation, reward processing, learning and memory, locomotion, food wanting, and dance communication [3,6,7]. Increasing evidence indicates that dopaminergic signaling constitutes a fundamental neuromodulatory system underlying diverse honeybee behaviors, including threat perception and defensive responses, social behavior, associative learning, and behavioral plasticity [8,9,10,11]. Collectively, these findings establish dopamine signaling as a central neural regulatory network that enables honeybees to adapt to dynamic environmental conditions and optimize behavioral decision-making. Building upon this framework, our previous study was the first to demonstrate that transient activation of dopaminergic signaling rapidly promotes food wanting in honeybees, establishing a neurobiological model in which dopamine regulates food wanting [3]. We subsequently showed that miR-375-3p modulates brain dopamine levels and food wanting by directly targeting DOPA decarboxylase (Ddc), a key enzyme responsible for dopamine biosynthesis, thereby revealing a previously unrecognized miRNA-mediated mechanism regulating dopamine biosynthesis and food wanting [12]. Nevertheless, despite the identification of miR-375-3p as a critical regulatory node linking dopamine biosynthesis to food wanting, the upstream long noncoding RNA (lncRNA) regulatory network regulating miR-375-3p and its downstream effects on food wanting in honeybees remain largely unknown.

In insect behavioral regulation, ncRNAs act as important post-transcriptional regulators and participate in diverse biological processes, including the modulation of neural activity and behavioral plasticity [13]. LncRNAs are transcripts longer than 200 nucleotides with little or no protein-coding potential and can regulate gene expression through multiple mechanisms, including chromatin modification, transcriptional regulation, modulation of RNA stability, and competing endogenous RNA (ceRNA)-mediated regulation [14]. In the ceRNA mechanism, lncRNAs can competitively bind microRNAs (miRNAs), thereby alleviating miRNA-mediated repression of target genes and modulating downstream gene expression [13]. Through this mechanism, lncRNAs can influence neural signaling, neuronal function, and behavioral regulation [13,15]. Accordingly, lncRNAs are increasingly recognized as important molecular mediators linking gene regulatory networks to behavior [16]. Studies in vertebrates have further implicated lncRNAs in the regulation of dopaminergic signaling and neuronal function, including through lncRNA–miRNA interactions [17,18]. As research has expanded beyond vertebrate systems, increasing attention has been directed toward the roles of lncRNAs in regulating neural function and behavior in invertebrates [19,20]. However, compared with mammals, functional studies of insect lncRNAs remain relatively limited. Current studies have largely focused on roles in development, reproduction, immunity, and environmental adaptation, whereas their functions in neurotransmitter regulation and behavioral modulation remain comparatively less explored [13,21]. To date, only a few studies have revealed functional links between lncRNAs and dopaminergic signaling. For example, Wang et al. reported that the locust lncRNA PAHAL regulates the expression of pale, a key enzyme involved in dopamine biosynthesis, through a feedback regulatory mechanism, thereby altering brain dopamine levels and modulating the plasticity of gregarious–solitary behavioral transitions [22]. Together, these findings highlight the emerging role of lncRNAs in neurobehavioral regulation and suggest that they represent an important regulatory layer linking gene expression to behavior.

Based on these findings, we hypothesized that lncRNAs act as upstream regulators of miR-375-3p and regulate dopamine biosynthesis through a ceRNA-mediated mechanism, thereby modulating food wanting in honeybees. In our previous study, full-length transcriptome analysis of honeybee brains identified 174 differentially expressed lncRNAs in honeybees exhibiting distinct food wanting states [23]. Building on these findings, the present study investigated whether lncRNAs participate in the miR-375-3p/*Ddc* regulatory pathway and contribute to the control of food wanting. We identified lnc6100 as a key upstream regulator of the miR-375-3p/*Ddc* axis and further characterized its role in dopamine-dependent food wanting. Our findings not only expand the molecular regulatory network underlying honeybee food wanting, but also provide the first evidence that an lncRNA-mediated ceRNA network functionally links noncoding RNA regulation, dopamine metabolism, and food wanting. Collectively, these findings provide new molecular insights into the neuromolecular regulation of food wanting and provide a theoretical basis for developing lncRNA-based strategies to improve honeybee foraging performance and colony health.

## 2. Materials and Methods

### 2.1. Insects

**Figure 1:**
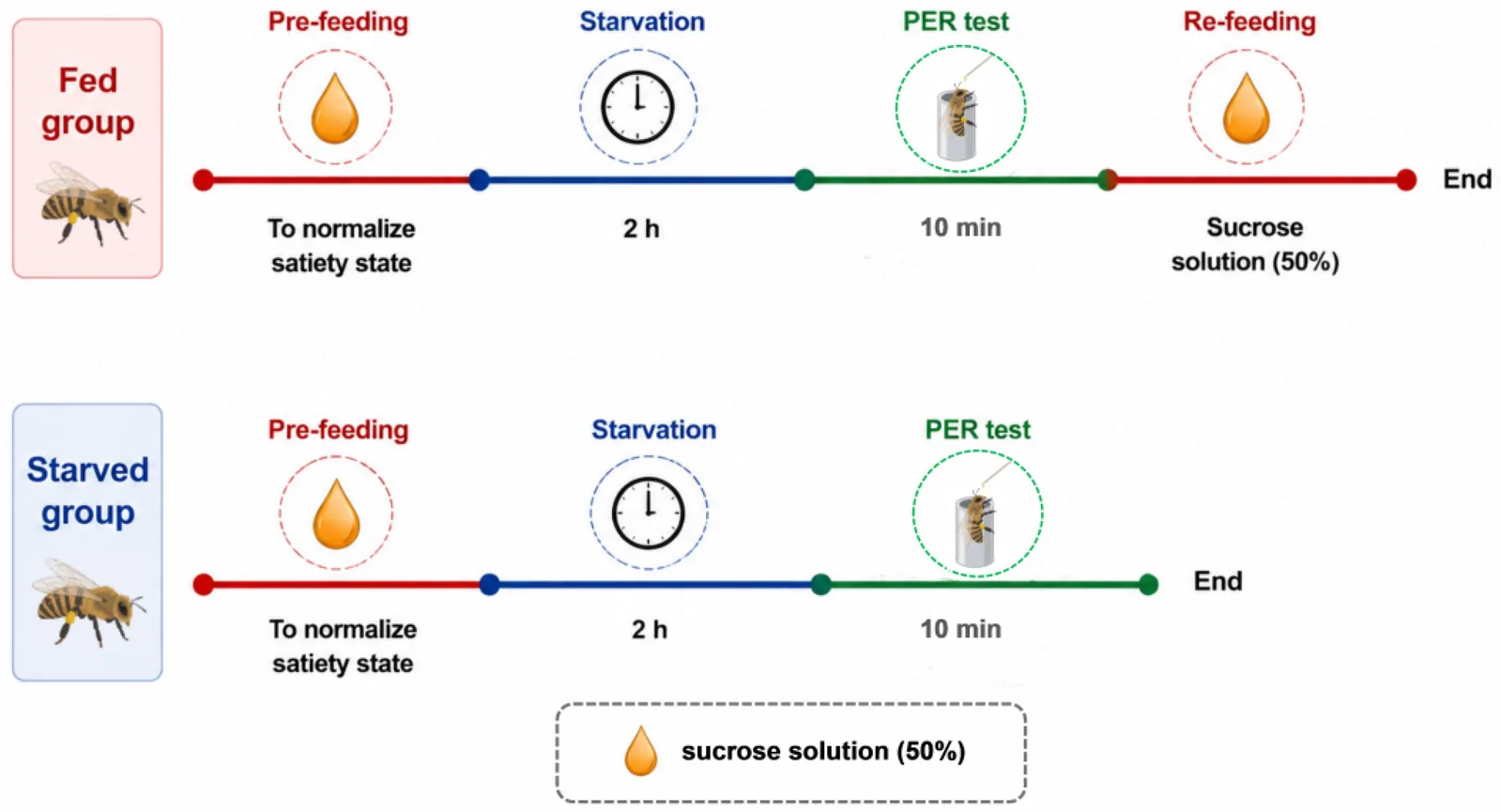
Experimental design and sampling scheme for fed (FB) and starved (SB) honeybees.

Honeybees (*Apis mellifera*) were obtained from three colonies maintained at Fuqing Honeybee Farm and managed at the College of Bee Science, Fujian Agriculture and Forestry University (Fuzhou, China). Colonies were maintained following standardized beekeeping protocols previously described by Wu et al. [12]. Before experimentation, all colonies were confirmed to be free of visible signs of disease and parasite infestation. One brood frame containing capped pupae was collected from each colony, yielding three brood frames in total. Newly emerged workers were collected daily for three consecutive days. Workers that emerged within 24 h were designated as 0-day-old bees, marked on the thorax, and returned to their source colonies. Worker honeybees at 23 days post-emergence were collected for subsequent experiments, and the experimental design for the fed and starved groups is illustrated in Figure 1.

### 2.2. Sucrose Solution Responsiveness Assay

Sucrose solution responsiveness was assessed following previously described protocols [24,25]. Briefly, the antennae were sequentially stimulated with increasing sucrose concentrations (0.1–50%, *w*/*w*), and the proboscis extension response (PER) was recorded at each concentration. The sucrose responsiveness score (SRS) was calculated as the number of sucrose concentrations eliciting a PER, with higher scores indicating greater sucrose responsiveness.

### 2.3. Sucrose Solution Consumption Assay

Sucrose solution consumption (SSC) was measured according to previously described methods [12]. The marked workers were returned to their original colonies after marking and were recaptured from the colonies at 23 days of age for the SSC assay. To standardize hunger levels, honeybees were starved for 2 h and then individually fed 50% (*w*/*w*) sucrose solution using a micropipette until satiation within 10 min. The total amount of sucrose solution consumed by each group was recorded, and the average consumption per honeybee was calculated.

### 2.4. Appetitive Olfactory Learning Assay

Appetitive olfactory learning was assessed following established classical conditioning protocols [3]. Odorants served as conditioned stimuli (CS), and 50% (*w*/*w*) sucrose solution served as the unconditioned stimulus (US). Honeybees underwent six conditioning trials consisting of rewarded CS+ and unrewarded CS− presentations, and learning performance was quantified using the acquisition score (ACQS).

### 2.5. Primer Design

Primers for mRNA and lncRNA detection were designed using Primer Premier 5.0 software based on the corresponding target sequences. miRNA-specific primers were designed using miRNA Design V1.01 (Vazyme Biotech, Nanjing, China). All primers were synthesized by Fuzhou SunYa Biotechnology Co., Ltd. (Fuzhou, China). The primer sequences are listed in Table S1.

### 2.6. Quantitative Real-Time Polymerase Chain Reaction (RT-qPCR)

Total RNA was extracted from the brain sample (*n* = 3 biological replicates; three bees per replicate) using TRIzol reagent (Thermo Fisher Scientific, Waltham, MA, USA), and 1 μg of RNA was reverse-transcribed into cDNA using the HiScript III RT SuperMix kit (Vazyme Biotech, Nanjing, China). The synthesized cDNA was used as the template for qPCR with ChamQ Blue Universal SYBR qPCR Master Mix (Vazyme Biotech, Nanjing, China). Amplification was performed on a Bio-Rad CFX Connect system with the following conditions: 95 °C for 30 s, followed by 40 cycles of 95 °C for 10 s and 60 °C for 30 s. Melting curve analysis was performed to confirm amplification specificity. For miRNA detection, reverse transcription and qPCR were performed using a miRNA-specific SYBR qPCR kit (Vazyme Biotech, Nanjing, China) according to the manufacturer’s instructions. *Actin* and U6 snRNA were used as internal references for mRNA/lncRNA and miRNA expression analyses, respectively. Relative expression levels were calculated using the 2^–ΔΔCT^ method. All reactions were performed with three technical replicates. The primers used in this study were designed using Primer Premier 5.0 software and synthesized by Fuzhou SunYa Biotechnology Co., Ltd. (Fuzhou, China). Their sequences are listed in Table S1.

### 2.7. Prediction and Network Analysis of lncRNA–miRNA Interactions

Potential interactions between candidate lncRNAs and miRNAs were predicted using the TargetScan (https://www.targetscan.org/) and miRanda (http://www.microrna.org/) databases based on miRNA sequence information available for arthropods. Candidate miRNA-binding sites within lncRNA sequences were identified according to sequence complementarity and predicted binding energy. The overlapping lncRNA–miRNA interaction pairs predicted by both databases were considered candidate interactions for subsequent analysis. The resulting lncRNA–miRNA interaction network was constructed and visualized using Cytoscape software (version 3.7.2).

### 2.8. In Vitro Dual-Luciferase Reporter Assay

To validate the interaction between lnc6100 and miR-375-3p, the fragment of lnc6100 containing the predicted miR-375-3p binding site was cloned into the psiCHECK-2 luciferase reporter vector (Promega, Madison, WI, USA) to generate the wild-type (lnc6100-Wt) construct. A mutant reporter (lnc6100-Mut) containing nucleotide substitutions within the predicted miR-375-3p binding site was generated by site-directed mutagenesis. All recombinant plasmids were verified by sequencing.

HEK293T cells were cultured until reaching 90–95% confluence and then co-transfected with lnc6100-Wt or lnc6100-Mut reporter plasmids and miR-375-3p mimics or the corresponding mimic negative control (NC) using Lipofectamine 3000 Transfection Reagent (Thermo Fisher Scientific, Waltham, MA, USA) according to the manufacturer’s instructions. After 24 h of incubation, luciferase activity was measured using the Dual-Luciferase Reporter Assay System (Promega, Madison, WI, USA). Relative luciferase activity was calculated as the ratio of *Renilla* luciferase activity to firefly luciferase activity, and the values were normalized to the corresponding control group.

### 2.9. RNAi for lncRNAs

Specific siRNAs targeting candidate lncRNAs (lnc6100, lnc3305155, lnc9171, and lnc1705568) and a non-targeting control siRNA (si-scramble) were designed and synthesized by Tsingke Biotechnology Co., Ltd. (Beijing, China) (Table S1). Each treatment group initially consisted of 150 worker honeybees (*n* = 150 per group). For in vivo knockdown, 2 μg of siRNA (1 μg/μL, 2 μL) was microinjected into the honeybee brain. The mortality rate following brain microinjection was approximately 5%, and only surviving bees were included in the subsequent analyses. At 24 h after injection, honeybees were subjected to RT-qPCR analysis to validate lncRNA knockdown efficiency and to behavioral assays to evaluate food wanting.

### 2.10. Behavioral Rescue Assay In Vivo

To determine whether lnc6100 regulates food wanting through the miR-375-3p/*Ddc* pathway, we performed brain microinjection-based rescue experiments. Each treatment group initially consisted of 150 worker honeybees (*n* = 150 per group). The mortality rate following brain microinjection was approximately 5%, and only surviving bees were included in the subsequent behavioral and molecular analyses. In the first rescue assay, honeybees were injected with si-lnc6100 (2 μg), followed by administration of 45 pmol miR-375-3p antagomir or the antagomir control (at-ck) 24 h later. In the second rescue assay, honeybees were initially injected with si-scramble and at-ck as control treatments and, 24 h later, received co-administration of si-lnc6100 (2 μg) and miR-375-3p antagomir (45 pmol). Three complementary behavioral assays were performed 24 h after the second injection to evaluate food wanting. Following behavioral assessments, honeybee brains were collected for analysis of *Ddc* expression levels and dopamine levels. Samples were immediately flash-frozen in liquid nitrogen and stored at −80 °C until further analysis. The related sequences are listed in Table S1.

### 2.11. Quantification of Dopamine in Honeybee Brains

Dopamine levels in honeybee brains were quantified using high-performance liquid chromatography coupled with electrochemical detection (HPLC-ECD), following a previously described method [3]. Briefly, individual honeybee brains were dissected, homogenized, and subjected to HPLC-ECD analysis using a Waters Arc HPLC system (Waters Corporation, Milford, MA, USA) equipped with a 3465 electrochemical detector. Dopamine content was determined using the external standard calibration method based on standard curves generated from serial dilutions of dopamine standards. The dopamine content in each sample was calculated according to the corresponding calibration curve.

### 2.12. Statistical Analysis

Statistical analyses were performed using GraphPad Prism 10.2.3, and data are presented as mean ± SEM. Statistical tests were selected according to data distribution: comparisons between two groups were conducted using Student’s *t*-test or Mann–Whitney *U* test, whereas multiple-group comparisons were performed using one-way ANOVA followed by Tukey’s post hoc test or Kruskal–Wallis test followed by Dunn’s post hoc test. Statistical significance was set at *p* < 0.05. Significance levels are indicated as ns, * *p* < 0.05, ** *p* < 0.01, *** *p* < 0.001, and **** *p* < 0.0001.

## 3. Results

### 3.1. Identification of Candidate DElncRNAs Associated with miR-375-3p

**Figure 2:**
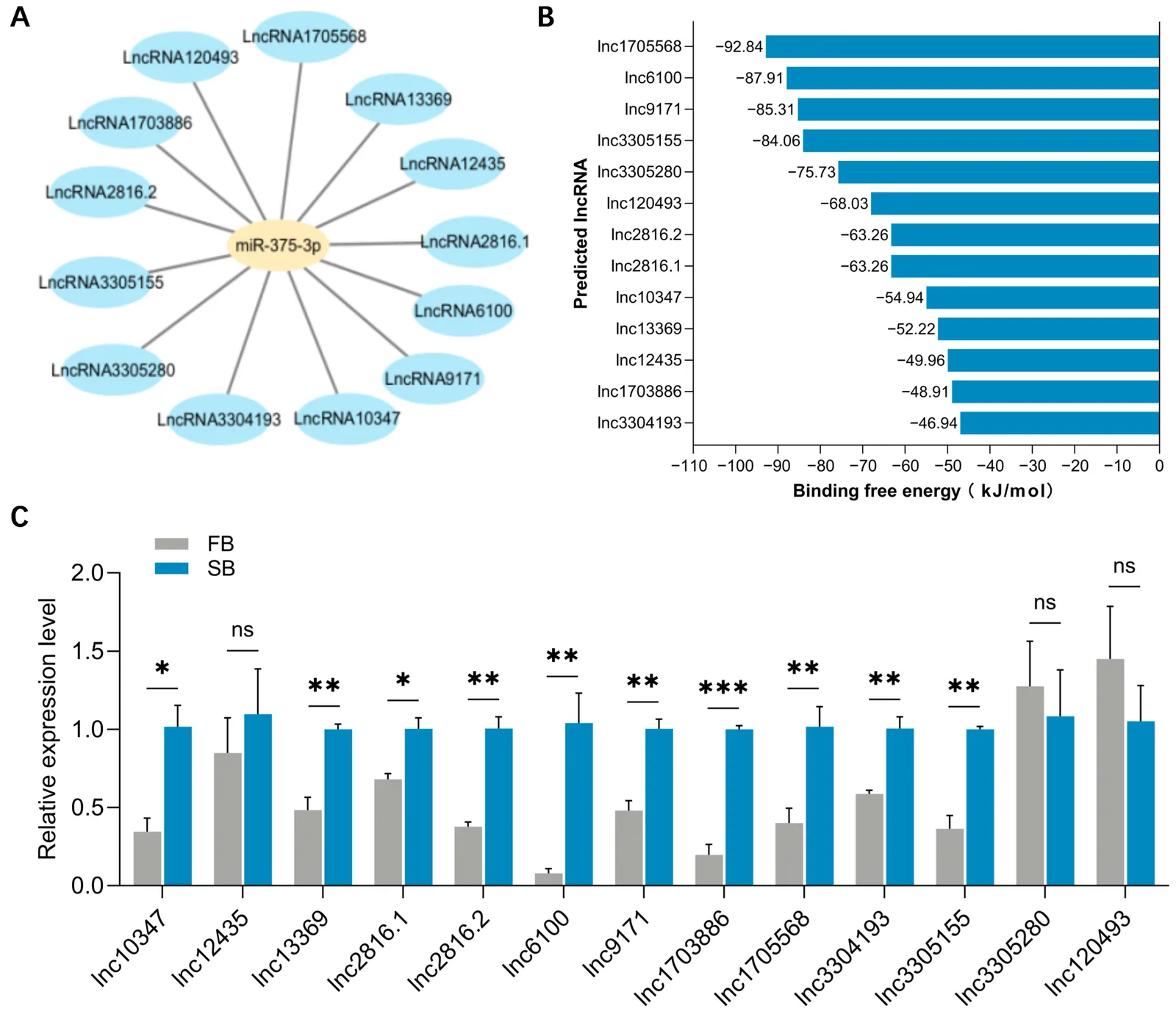
Screening and validation of candidate lncRNAs associated with miR-375-3p. (**A**) Schematic representation of the predicted lncRNA–miR-375-3p regulatory network. (**B**) Predicted interaction energies between miR-375-3p and candidate lncRNAs. (**C**) Expression validation of candidate lncRNAs in honeybee brains by RT-qPCR. Data are presented as mean ± SEM. * *p* < 0.05; ** *p* < 0.01; *** *p* < 0.001 and ns, not significant.

To identify potential lncRNAs involved in the miR-375-3p-mediated regulatory network, we integrated sequencing data with bioinformatic analysis to predict lncRNAs containing potential miR-375-3p binding sites. Based on these predictions, we constructed a lncRNA–miR-375-3p interaction network and identified 13 candidate lncRNAs predicted to interact with miR-375-3p (Figure 2A).

To further prioritize these candidates, we compared the predicted binding energies between miR-375-3p and the 13 lncRNAs (Table S2). Four candidates showed relatively stronger predicted binding capacities, with binding energies lower than −83.68 kJ/mol (−20 kcal/mol) (Figure 2B), indicating potentially stable interactions with miR-375-3p.

We next validated the expression levels of the candidate lncRNAs identified by RNA-seq using RT-qPCR in honeybee brains (Table S3). RT-qPCR analysis revealed differential expression patterns among the 13 candidate lncRNAs. Three candidates, including lnc12345, lnc3305280, and lnc120493, showed no significant expression changes and were excluded from subsequent functional evaluation (Figure 2C). By integrating the predicted binding potential with miR-375-3p and the expression characteristics of candidate lncRNAs, we selected lnc6100, lnc9171, lnc1705568, and lnc3305155 for further functional characterization.

### 3.2. lnc6100 Regulates Honeybee Food Wanting

**Figure 3:**
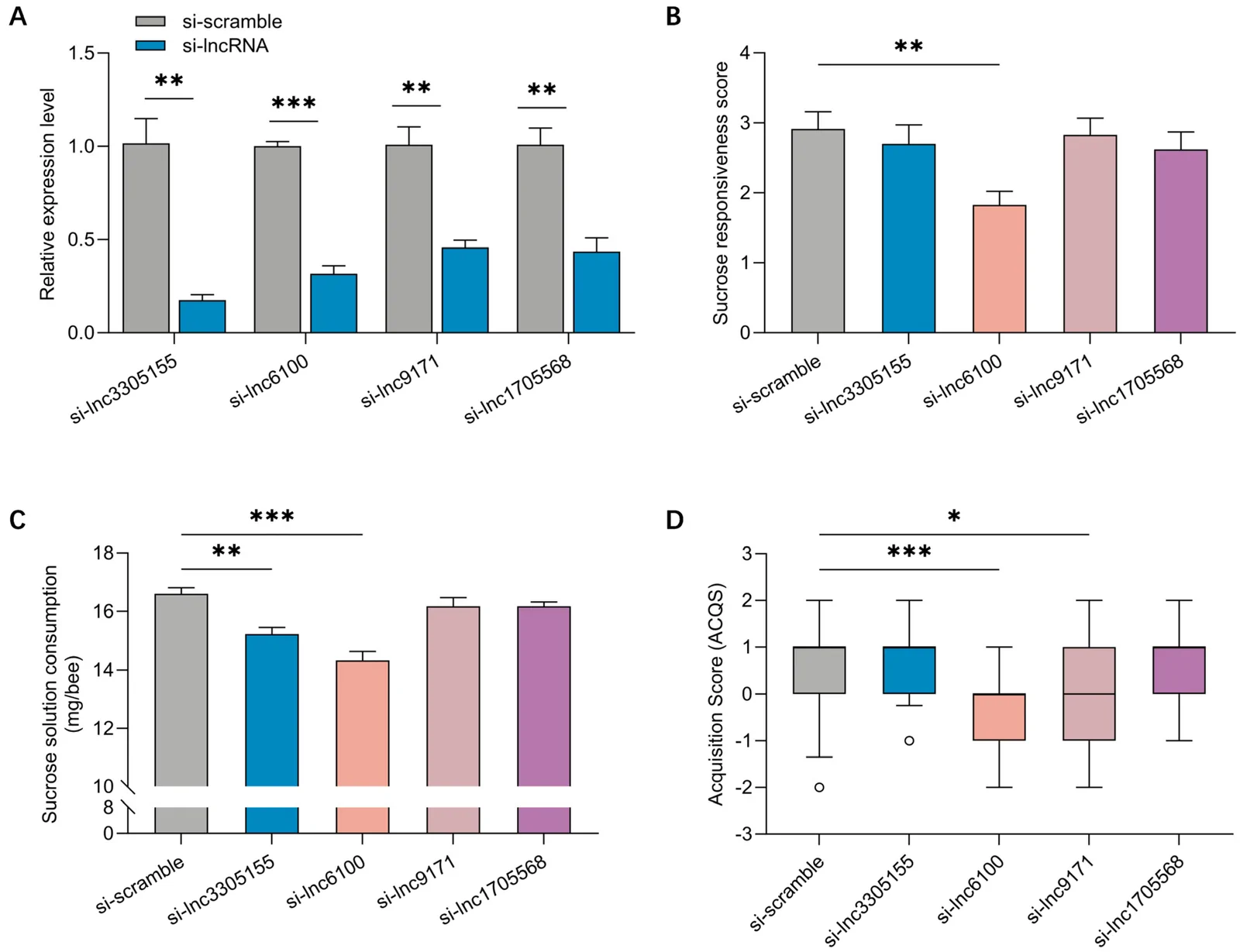
Effects of candidate lncRNA knockdown on honeybee food wanting. (**A**) Knockdown efficiency of four candidate lncRNAs (lnc1705568, lnc6100, lnc9171, and lnc3305155) after siRNA injection was validated by RT-qPCR. (**B**) Effects of lncRNA knockdown on sucrose responsiveness score (*n* ≥ 30). (**C**) Effects of lncRNA knockdown on sucrose solution consumption (*n* ≥ 30). (**D**) Effects of lncRNA knockdown on appetitive olfactory learning acquisition score (*n* ≥ 30). Data are presented as mean ± SEM. * *p* < 0.05; ** *p* < 0.01; *** *p* < 0.001.

To investigate whether the four candidate lncRNAs (lnc1705568, lnc6100, lnc9171, and lnc3305155) regulate honeybee food wanting, we performed functional validation using RNA interference. We injected 23-day-old worker honeybees with lncRNA-specific siRNAs, and RT-qPCR analysis confirmed significant reductions in the expression of all four candidate lncRNAs, indicating efficient knockdown (Figure 3A).

We subsequently evaluated the effects of silencing these four candidate lncRNAs on food wanting using three behavioral assays, including sucrose responsiveness score (SRS), sucrose solution consumption (SSC), and appetitive olfactory learning acquisition score (ACQS). Compared with the si-scramble control group, knockdown of the four candidate lncRNAs produced distinct effects on food wanting. Among the four candidate lncRNAs, only lnc6100 exhibited significant effects across all three behavioral assays (Figure 3B–D). Specifically, knockdown of lnc6100 significantly decreased the sucrose responsiveness score from 2.912 to 1.829 (Figure 3B), reduced sucrose solution consumption from 16.6 ± 0.1 mg to 14.3 ± 0.1 mg (Figure 3C), and impaired appetitive olfactory learning acquisition, with ACQS decreasing from 0.719 to −0.206 (Figure 3D). These results indicate that lnc6100 positively regulates food wanting in honeybees. In comparison, knockdown of lnc3305155 significantly reduced sucrose solution consumption from 16.6 ± 0.1 mg to 15.2 ± 0.1 mg (Figure 3C), but did not affect sucrose responsiveness or appetitive olfactory learning acquisition (Figure 3B–D). Similarly, silencing of lnc9171 significantly impaired appetitive olfactory learning acquisition, with ACQS decreasing from 0.719 to 0.032 (Figure 3D), whereas no significant changes were observed in sucrose responsiveness or sucrose solution consumption (Figure 3B,C). Furthermore, knockdown of lnc1705568 did not produce significant effects in any of the tested behavioral assays (Figure 3B–D).

Collectively, our results demonstrate that among the four candidate lncRNAs, lnc6100 exhibited regulatory effects across all three behavioral assays, suggesting that lnc6100 functions as a key regulator of food wanting in honeybees.

### 3.3. lnc6100 Interacts with miR-375-3p and Modulates Ddc-Mediated Dopamine Synthesis

**Figure 4:**
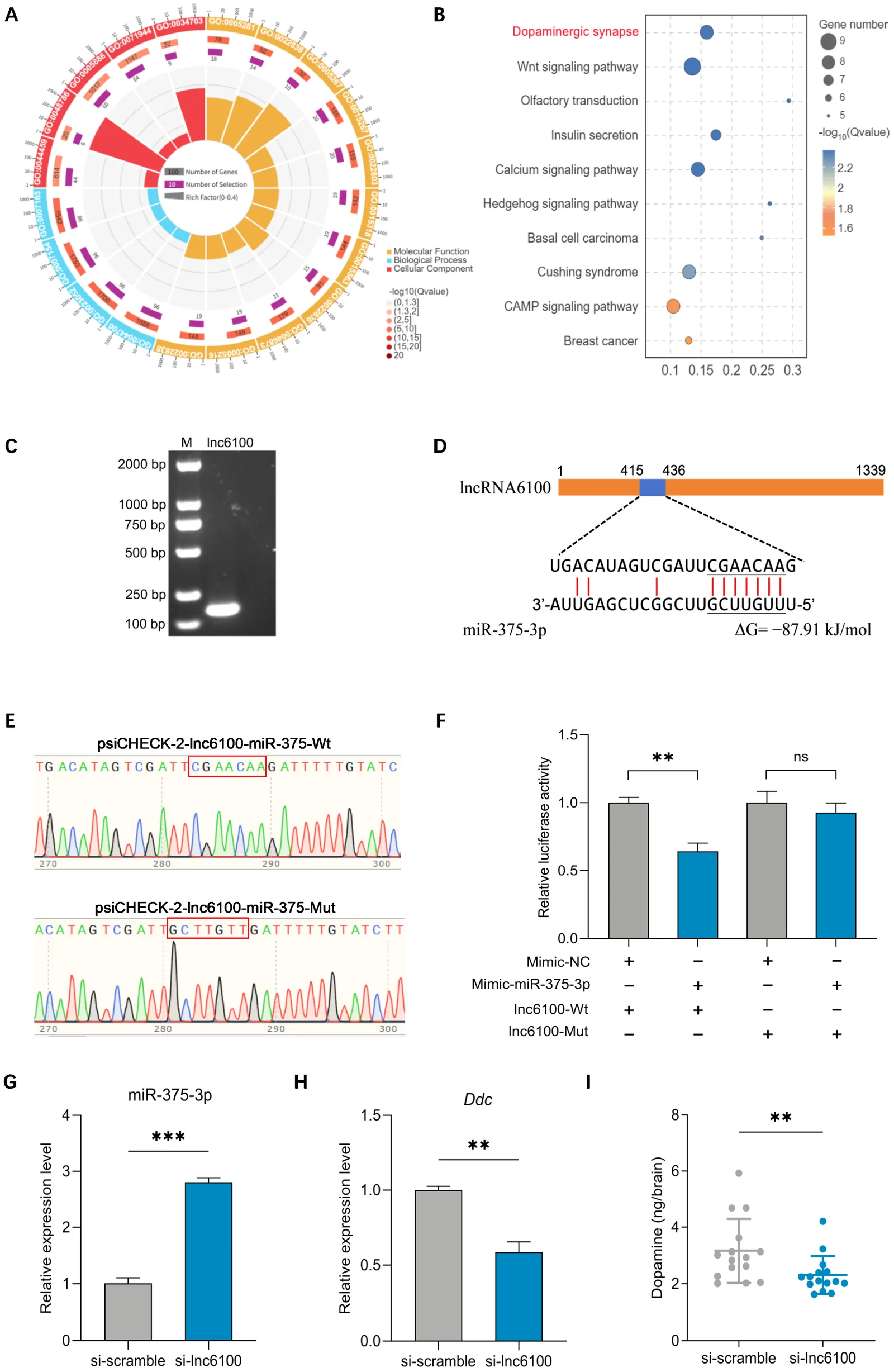
Molecular characterization of lnc6100 and its potential role in miR-375-3p/*Ddc*-mediated dopamine synthesis. (**A**) GO enrichment analysis of differentially expressed mRNAs associated with lnc6100. (**B**) KEGG pathway enrichment analysis of differentially expressed mRNAs associated with lnc6100. The dopaminergic synapse pathway is highlighted in red. (**C**) Agarose gel electrophoresis analysis of the amplified lnc6100 fragment. (**D**) Schematic diagram of the interaction between lnc6100 and miR-375-3p. The predicted binding free energy is −87.91 kJ/mol. (**E**) Sanger sequencing confirmation of the psiCHECK-2-lnc6100-miR-375-Wt and psiCHECK-2-lnc6100-miR-375-Mut reporter constructs. Red boxes indicate the miR-375-3p binding site in the wild-type construct and the corresponding mutated sequence in the mutant construct. (**F**) Dual-luciferase reporter assay for validation of the interaction between lnc6100 and miR-375-3p. (**G**) Expression level of miR-375-3p after lnc6100 knockdown. (**H**) Expression level of *Ddc* after lnc6100 knockdown. (**I**) Dopamine levels in honeybee brains following lnc6100 knockdown (*n* = 15). Data are presented as mean ± SEM. ** *p* < 0.01; *** *p* < 0.001 and ns, not significant.

To investigate the potential molecular mechanism underlying the regulatory function of lnc6100, we performed functional enrichment analyses of the differentially expressed mRNAs associated with lnc6100. Gene Ontology (GO) analysis showed that lnc6100-associated differentially expressed mRNAs were mainly enriched in signaling-related biological processes, including signaling, cell communication, and signal transduction, as well as molecular functions related to ion transport, such as cation channel activity, potassium ion channel activity, and transmembrane transporter activity (Figure 4A). Kyoto Encyclopedia of Genes and Genomes (KEGG) pathway enrichment analysis further revealed that these genes were involved in multiple signaling pathways, including the dopaminergic synapse pathway, Wnt signaling pathway, olfactory transduction, insulin secretion, calcium signaling pathway, Hedgehog signaling pathway, cAMP signaling pathway (Figure 4B). Notably, enrichment in the dopaminergic synapse pathway prompted us to investigate whether lnc6100 participates in dopamine-related regulation. Given our previous finding that miR-375-3p regulates honeybee food wanting through the *Ddc*-mediated dopamine synthesis pathway, we hypothesized that lnc6100 might act upstream of miR-375-3p.

A specific fragment of lnc6100 was amplified, and the expected PCR product was confirmed by agarose gel electrophoresis (Figure 4C). Subsequently, bioinformatic prediction identified a putative miR-375-3p binding site within the lnc6100 sequence (Figure 4D). The wild-type lnc6100 fragment and its mutant counterpart containing nucleotide substitutions within the predicted miR-375-3p binding site were then cloned into the psiCHECK-2 vector. Sanger sequencing confirmed the accuracy of the recombinant plasmids psiCHECK-2-lnc6100-miR-375-Wt and psiCHECK-2-lnc6100-miR-375-Mut (Figure 4E). Dual-luciferase reporter assays were performed in HEK293T cells to verify the interaction between lnc6100 and miR-375-3p. The results showed that miR-375-3p mimics significantly reduced the luciferase activity of the psiCHECK-2-lnc6100-miR-375-Wt reporter (*p* = 0.0077). However, when the predicted miR-375-3p binding site within lnc6100 was mutated, miR-375-3p mimics did not significantly affect luciferase activity of the psiCHECK-2-lnc6100-miR-375-Mut reporter (*p* > 0.05) (Figure 4F). These results support a direct interaction between lnc6100 and miR-375-3p through the predicted binding site.

To determine whether this interaction contributes to the regulation of the miR-375-3p/*Ddc* pathway, we examined miR-375-3p, *Ddc* expression and dopamine levels following lnc6100 knockdown. Compared with the si-scramble control group, silencing of lnc6100 significantly increased miR-375-3p expression (Figure 4G). In contrast, knockdown of lnc6100 significantly reduced the expression level of *Ddc* (Figure 4H), consistent with the established regulatory relationship between miR-375-3p and *Ddc*. Furthermore, lnc6100 silencing significantly decreased dopamine levels in honeybee brains, with approximately 27% reduction compared with the control group (Figure 4I). Together, these findings support a role for lnc6100 in modulating dopamine synthesis through the miR-375-3p/*Ddc* pathway.

### 3.4. The lnc6100–miR-375-3p–Ddc Axis Controls Honeybee Food Wanting

**Figure 5:**
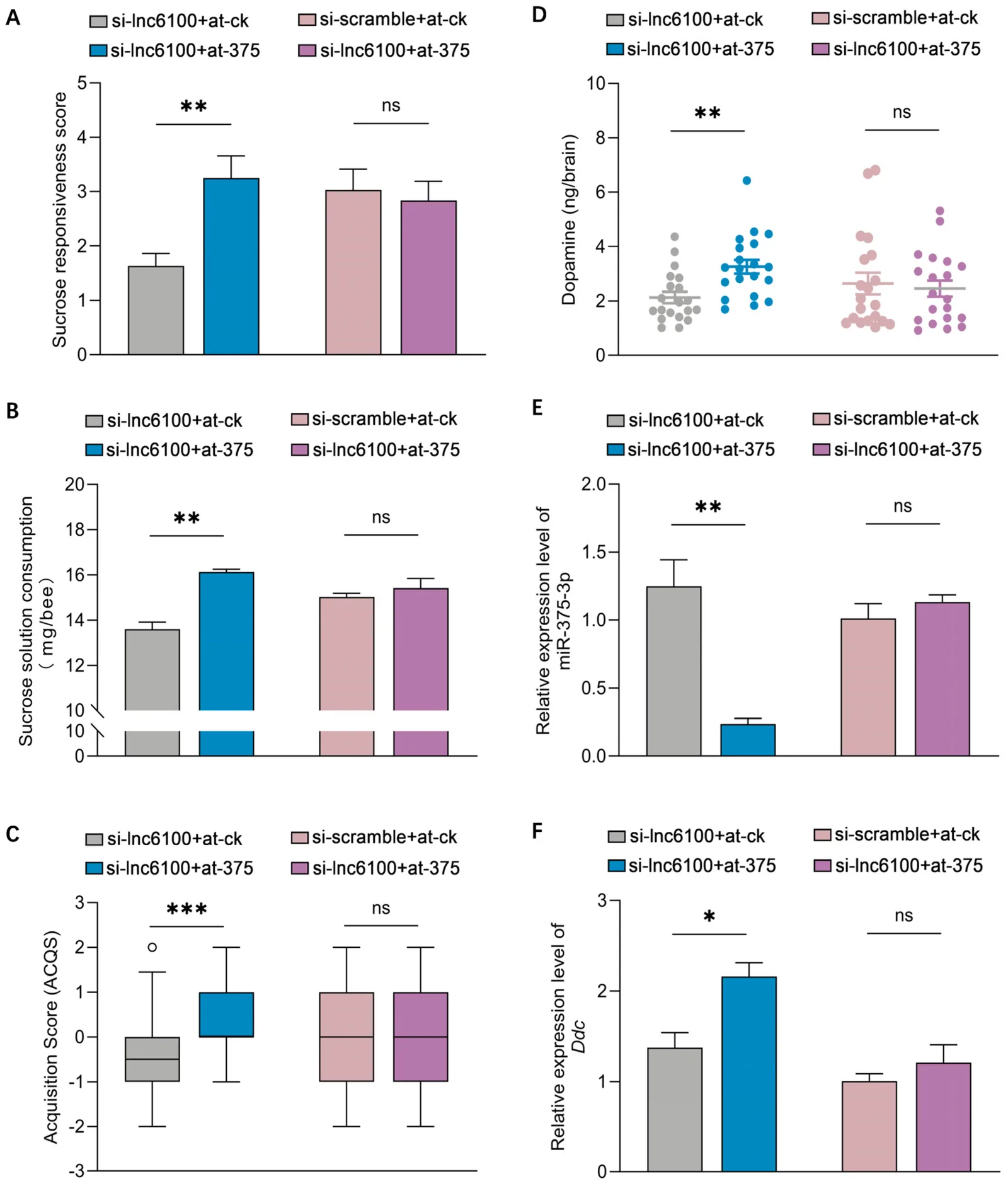
(**A**–**C**) Food wanting following miR-375-3p inhibition after lnc6100 knockdown and in the combined interference experiment, as assessed by sucrose responsiveness score (**A**), sucrose solution consumption (**B**), and appetitive olfactory learning acquisition score (**C**). (**D**) Brain dopamine levels following miR-375-3p inhibition after lnc6100 knockdown and in the combined interference experiment. (**E**,**F**) Expression levels of miR-375-3p (**E**) and *Ddc* (**F**) following miR-375-3p inhibition after lnc6100 knockdown and in the combined interference experiment. Data are presented as mean ± SEM ((**A**–**C**), *n* ≥ 30; (**D**), *n* = 20). * *p* < 0.05; ** *p* < 0.01; *** *p* < 0.001 and ns, not significant.

To determine whether lnc6100 regulates food wanting through miR-375-3p, we first examined whether inhibition of miR-375-3p could rescue the reduction in food wanting induced by lnc6100 silencing. Inhibition of miR-375-3p alleviated the impaired food wanting caused by lnc6100 silencing (Figure 5A–C). Recovery was observed across all three behavioral assays after antagomir-375-3p treatment. The sucrose responsiveness score increased from 1.633 to 3.250, sucrose solution consumption increased from 13.6 ± 0.1 mg to 16.1 ± 0.1 mg, and appetitive olfactory learning acquisition was improved, with the ACQS increasing from −0.50 to 0.34 (Figure 5A–C). Furthermore, inhibition of miR-375-3p significantly restored dopamine levels in honeybee brains, resulting in an approximately 38% increase compared with the control group (Figure 5D). Consistently, molecular analysis showed that antagomir-375-3p treatment reduced miR-375-3p expression and increased *Ddc* expression (Figure 5E,F), supporting the regulatory relationship between miR-375-3p and *Ddc*.

To further determine whether the lnc6100/miR-375-3p regulatory axis regulates food wanting through *Ddc*, we performed a combined interference experiment involving lnc6100 knockdown and miR-375-3p inhibition. Under these conditions, inhibition of miR-375-3p failed to rescue the reduced food wanting induced by lnc6100 knockdown (Figure 5A–C). Consistent with these behavioral findings, brain dopamine levels also showed no significant change following the combined interference (Figure 5D). RT-qPCR analysis further showed that combined interference did not significantly alter the expression levels of miR-375-3p or *Ddc* (Figure 5E,F). These findings further support a critical role for *Ddc* as a downstream effector of the lnc6100–miR-375-3p regulatory axis in regulating honeybee food wanting.

## 4. Discussion

**Figure 6:**
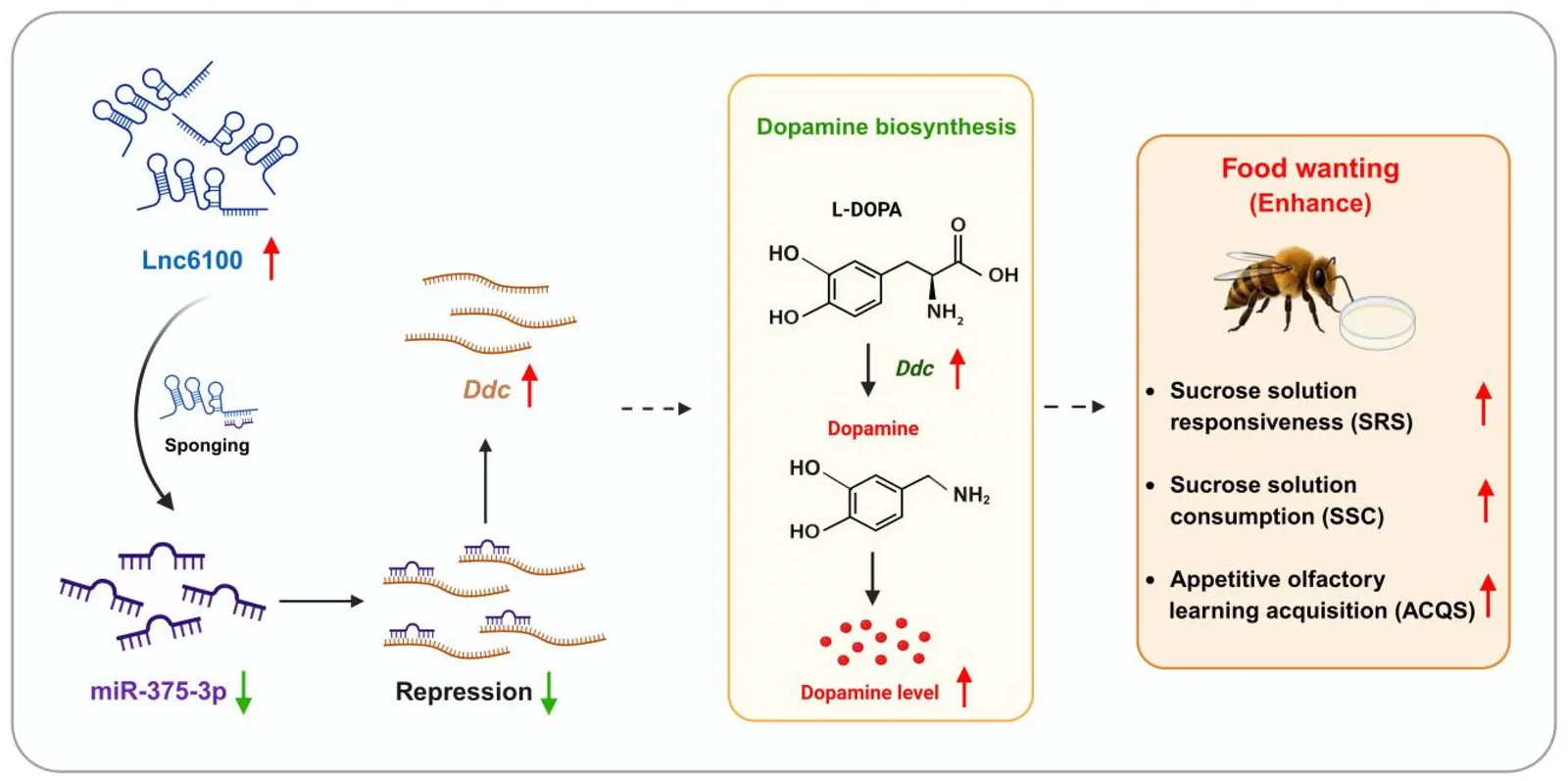
Proposed model of lnc6100-mediated regulation of dopamine-dependent food wanting in honeybees. Increased lnc6100 levels facilitate its interaction with miR-375-3p, thereby reducing miR-375-3p-mediated repression of *Ddc*. The resulting increase in *Ddc* expression promotes dopamine synthesis and enhances food wanting.

Food wanting represents a complex motivational state that emerges from the integration of internal physiological conditions, sensory perception, and neural reward systems [26]. Although extensive studies have revealed the central role of neuromodulators, particularly dopamine, in regulating motivation and reward-related behaviors [11,27], the upstream molecular mechanisms that modulate dopamine-dependent behavioral plasticity remain incompletely understood. Here, we identify a lncRNA-mediated regulatory mechanism that links noncoding RNA networks to dopamine-dependent food wanting in honeybees. Our findings reveal that lnc6100 regulates honeybee food wanting through the lnc6100–miR-375-3p–*Ddc* regulatory axis, establishing a direct link between noncoding RNA regulation, dopamine biosynthesis, and motivational behavior (Figure 6). This discovery highlights noncoding RNA networks as an important regulatory layer linking dopamine signaling to food wanting and provides new insights into the molecular mechanisms underlying adaptive foraging decisions in honeybees.

Previous studies have established miRNAs as important post-transcriptional regulators of neuronal function and behavior [28,29]. Our previous work demonstrated that miR-375-3p suppresses honeybee food wanting by targeting *Ddc*, thereby limiting dopamine synthesis [12]. In the present study, we identify lnc6100 as an upstream regulatory component that modulates miR-375-3p activity. Among multiple candidate lncRNAs associated with miR-375-3p, only lnc6100 consistently altered sucrose responsiveness, sucrose consumption, and appetitive olfactory learning acquisition, three complementary behavioral readouts that collectively represent honeybee food wanting. The convergence of these behavioral effects identifies lnc6100 as a central regulator of the food wanting state in honeybees.

Interestingly, the candidate lncRNAs showed different effects across the three behavioral assays. Knockdown of lnc6100 consistently affected sucrose responsiveness, sucrose solution consumption, and appetitive olfactory learning acquisition, whereas knockdown of lnc3305155 and lnc9171 produced significant effects only on sucrose solution consumption and appetitive olfactory learning acquisition, respectively. These results suggest that different noncoding RNAs may exert selective effects on individual behavioral traits. This observation is consistent with previous studies showing that different noncoding RNAs can regulate behavioral traits through distinct molecular pathways in honeybees [30,31,32]. For example, previous studies have reported that multiple miRNAs are involved in regulating sucrose solution responsiveness in honeybees, with *ame-let-7* enhancing solution responsiveness through *Amdop2* and *ame-miR-279a* also contributing to solution responsiveness in forager honeybees [31,32]. Several lncRNAs, including TCONS_00356023, TCONS_00357367, and TCONS_00159909, have been implicated in the regulation of foraging behavioral transitions [30]. Together, these findings suggest that a given behavioral trait may be regulated by multiple noncoding RNAs through distinct molecular pathways rather than by a single regulatory mechanism. In contrast, the consistent effects of lnc6100 across three complementary behavioral assays provide convergent evidence for its role in regulating food wanting, strengthening the inference that lnc6100 is a regulator of this motivational state. Increasing evidence indicates that lncRNAs are not merely transcriptional by-products but represent important regulatory layers involved in physiological processes and behavioral regulation in insects. For example, Zhang et al. demonstrated that the locust lncRNA PAHAL modulates behavioral plasticity through a feedback regulation of dopamine biosynthesis, revealing a functional connection between lncRNA regulation, neurotransmitter metabolism, and behavioral adaptation [22]. In addition, the neuronal lncRNA LRG in *Drosophila* regulates locomotion by interacting with Synaptotagmin 1, a key protein involved in synaptic vesicle release, thereby modulating neural transmission and behavioral outputs [33]. The findings suggest that lncRNAs represent an important regulatory layer connecting molecular regulatory networks with neural behaviors.

lncRNAs can regulate gene expression through multiple mechanisms, among which interaction with miRNAs and modulation of their activity represent a well-established post-transcriptional regulatory mode, commonly described as the ceRNA mechanism [34,35]. Beyond serving as competing binding molecules for miRNAs, some lncRNAs can also function as precursors for miRNA biogenesis. In addition, lncRNAs may influence miRNA expression by affecting their transcription, processing, or stability [36,37,38]. These diverse regulatory modes highlight that lncRNAs are not merely transcriptional by-products but can function as upstream regulators within complex post-transcriptional regulatory networks. In the present study, dual-luciferase reporter assays together with expression analyses provided evidence for a functional interaction between lnc6100 and miR-375-3p. Silencing lnc6100 increased miR-375-3p expression while reducing the expression of its downstream target *Ddc*, suggesting that lnc6100 may buffer the inhibitory activity of miR-375-3p toward *Ddc*. These findings support a regulatory relationship between lnc6100 and the miR-375-3p/*Ddc* axis and suggest that this interaction may contribute to the regulation of food wanting.

The functional significance of lncRNA–miRNA regulatory networks has also begun to emerge in honeybees and related biological systems. Recent studies have revealed that such regulatory interactions can participate in diverse physiological and pathological processes. The lncRNA6470/ace-miR-750-y axis has been implicated in the regulation of *AcPP2A* expression, immune responses, and larval survival in the eastern honey bee during fungal infection. Similarly, lncRNA6140 from the chalkbrood pathogen *Ascosphaera apis* participates in the milR5658-x–ATPase regulatory axis, and interference with lncRNA6140 and milR5658-x affects ATPase expression and chalkbrood development [16,39]. These findings suggest that lncRNA–miRNA regulatory networks can exert diverse biological functions in honeybee-associated systems. To further test whether miR-375-3p functions downstream of lnc6100, we performed two complementary rescue experiments. Inhibition of miR-375-3p following lnc6100 knockdown restored the impaired food wanting, whereas simultaneous interference with lnc6100 and miR-375-3p failed to produce the expected reduction in food wanting under the combined intervention condition. Given our previous finding that dopamine is a key neurochemical mediator of food wanting [3], we next asked whether dopamine also mediates the effects of the lnc6100–miR-375-3p regulatory interaction. Our results showed that inhibition of miR-375-3p following lnc6100 knockdown significantly increased brain dopamine levels. Collectively, these findings support a hierarchical regulatory model in which lnc6100 maintains *Ddc* expression by limiting miR-375-3p activity, thereby promoting dopamine synthesis and ultimately influencing food wanting.

Because extra-hive conditions may elicit fear, escape, and defensive behaviors, while dopamine also contributes to reward, aggression, learning, and memory [8,40,41], the behavioral effects observed here may not be entirely specific to food wanting. Validation under natural hive conditions will therefore be important to determine whether this regulatory pathway specifically modulates food wanting or also contributes to other dopamine-dependent behavioral processes.

Overall, our study identifies lnc6100 as an upstream regulator of honeybee food wanting and supports a lnc6100–miR-375-3p–*Ddc* regulatory axis connecting noncoding RNA regulation with dopamine biosynthesis. These findings provide a molecular framework linking noncoding RNA regulation to dopamine-dependent food wanting and offer insights into the molecular basis of motivational processes that contribute to foraging behavior in honeybees.

## 5. Conclusions

In conclusion, our study establishes a lnc6100–miR-375-3p–*Ddc* regulatory axis that links noncoding RNA regulation to dopamine biosynthesis and food wanting in honeybees. lnc6100 promotes food wanting by restraining miR-375-3p-mediated repression of *Ddc*, thereby supporting dopamine synthesis. These findings reveal a previously uncharacterized noncoding RNA regulatory mechanism underlying a dopamine-dependent motivational state and provide new insights into the molecular basis of food wanting and behavioral plasticity in honeybees.

## Data Availability

The original contributions presented in the study are included in the article; further inquiries can be directed to the corresponding authors.

## Supplementary Materials

The following supporting information can be downloaded at: https://www.mdpi.com/article/10.3390/biom16101452/s1, Table S1: List of primers. Table S2: Predicted binding energies between candidate lncRNAs and miR-375-3p. Table S3: RNA-seq results for candidate lncRNAs associated with miR-375-3p.
