# Supplementary material for: A lnc6100-Mediated ceRNA Network Regulates Dopamine-Dependent Food Wanting in Honeybees
Source: Biomolecules. 2026 Oct 5;16(10):1452. doi: 10.3390/biom16101452 (PMC13702480; doi:10.3390/biom16101452)
Supplement: Supplementary file 1 [file biomolecules-16-01452-s001.zip › Table S1.pdf]

Table S1. List of primers

Primer	Primer sequence（5′-3′）	Purpose
Lnc10347-F	GTGCGGACAATGGTCAGTAAT	qRT-PCR
Lnc10347-R	CCTCTTCGGTCTTCCTCCTT	
Lnc12345-F	GCGTGAGAGCAGAAATGAAAC	
Lnc12345-R	TGGAGTAAGGTGTCAGTTGGA	
Lnc13369-F	GCGGGAAGAGGAGGATTCAA	
Lnc13369-R	CGTGGCGTGATCGTAAGTTC	
Lnc2861.1-F	GCAATCGTTCACTTATCCTCCAA	
Lnc2861.1-R	GGCAATCTCCTCCCTCTCTAC	
Lnc2861.2-F	TGCCGGTACAAGTTCTCGAA	
Lnc2861.2-R	CCGAACGAAAGTTTGTTTACGAAT	
Lnc6100-F	AGGAATTGGTGAAGGGTGTTT	
Lnc6100-R	GTAGACATCATCGCAGTACATCA	
Lnc9171-F	TTCCACCACGCTATCCATATCT	
Lnc9171-R	GTATTCGTCCCGTTTCGTTATCAT	
Lnc1703886-F	GCAAGCAGTGGAAGAGGTTAC	
Lnc1703886-R	AGATGGCGTATGCGTGTAGA	
Lnc1705568-F	AATGATGAAGATCCGAACGAAGG	
Lnc1705568-R	GACGATTGCGATGCCAGTTA	
Lnc3304193-F	GGATGATGGACGAGGCACTA	
Lnc3304193-R	GATGAACAATGAGGGACACGATT	
Lnc3305155-F	ACCAATAAGCGAGCGAGGAA	
Lnc3305155-R	AGGACAATGAAGCCGCACTA	
Lnc3305280-F	CCGAACTGTCCGTACCTCTT	
Lnc3305280-R	ACACACATTCCTCCGCATTG	
Lnc120493-F	TCCTCGTCCACTGTATATCATTCA	
Lnc120493-R	TTCTAACCATTCCTTCGTGTTGTT	
miR-375-3p-F	GCGTTTGTTCGTTCGGCTC	
miR-375-3p-R	AGTGCAGGGTCCGAGGTATT	
Actin-F	CCTAGCACCATCCACCATGAA	
Actin-R	GAAGCAAGAATTGACCCACCAA	
Ddc-F	GGATGTACGGCGTGGAGAAT	
Ddc-R	CAGACGAGCCCTAGAACGAC	
U6-F	GTTAGGCTTTGACGATTTCG	

U6-R	GGCATTTCTCCACCAGGTA	
miR-375-3p-F	GCGTTTGTTCGTTCGGCTC	
miR-375-3p-R	AGTGCAGGGTCCGAGGTATT	
si-lnc3305155	GGAGAAAGAUAUAAAUAUA<br>UAUAUUUAUAUCUUUCUCC	lncRNA knockdown
si-lnc3305155	CGCGGAAAUCCUAUGUUAA<br>UUAACAUAGGAUUUCCGCG	
si-lnc6100	CAUGUUCUAUUGACAUAGUCG<br>CGACUAUGUCAAUAGAACAUG	
si-lnc9171	GGAUUUGGUAAACGAUUGAAA<br>UUUCAAUCGUUUACCAAAUCC	
si-scramble	UUCUCCGAACGUGUCACGU<br>ACGUGACACGUUCGGAGAA	
antagomir-miR-375-3p	UAACUCGAGCCGAACGAACAAA	miR-375-3p inhibition
antagomir-ck	UCACAACCUCCUAGAAAGAGUAGA	Negative control

*F: forward primer; R: reverse primer
